# Methyl-Cantharidimide (MCA) Has Anticancer Efficacy in ABCB1- and ABCG2-Overexpressing and Cisplatin Resistant Cancer Cells

**DOI:** 10.3389/fonc.2020.00932

**Published:** 2020-06-26

**Authors:** Yi-Dong Li, Yong Mao, Xing-Duo Dong, Zi-Ning Lei, Yuqi Yang, Lizhu Lin, Charles R. Ashby, Dong-Hua Yang, Ying-Fang Fan, Zhe-Sheng Chen

**Affiliations:** ^1^Department of Pharmaceutical Sciences, College of Pharmacy and Health Sciences, St. John's University, Queens, NY, United States; ^2^Department of Oncology, Affiliated Hospital of Jiangnan University, Wuxi, China; ^3^Cancer Center, The First Affiliated Hospital of Guangzhou University of Chinese Medicine, Guangzhou, China; ^4^Department of Hepatobiliary Surgery, Zhujiang Hospital, Southern Medical University, Guangzhou, China

**Keywords:** methyl-cantharidimide (MCA), multidrug resistance (MDR), ABCB1, ABCG2, cisplatin resistance, cell apoptosis, unc-5 netrin receptor B (UNC5B)

## Abstract

In this study, we investigated the efficacy of methyl-cantharidimide (MCA), a cantharidin (CTD) analog, as an anticancer drug, in cancer cells overexpressing either ABCB1 or ABCG2 transporters and in cisplatin-resistant cancer cells. The results indicated that: (i) MCA was efficacious in the ABCB1-overexpressing cell line, KB-C2, and the ABCB1-gene-transfected cell line, HEK293/ABCB1 (IC50 from 6.37 to 8.44 mM); (ii) MCA was also efficacious in the ABCG2-overexpressing cell line, NCI-H460/MX20, and the ABCG2-gene-transfected cell lines, HEK293/ABCG2-482-R2, HEK293/ABCG2-482-G2, and the HEK293/ABCG2-482-T7 cell lines (IC50 from 6.37 to 9.70 mM); (iii) MCA was efficacious in the cisplatin resistant cancer cell lines, KCP-4 and BEL-7404/CP20 (IC50 values from 7.05 to 8.16 mM); (iv) MCA (up to 16 mM) induced apoptosis in both BEL-7404 and BEL-7404/CP20 cancer cells; (v) MCA arrested both BEL-7404 and BEL-7404/CP20 cancer cells in the G0/G1 phase of the cell cycle; (vi) MCA (8 mM) upregulated the expression level of the protein, unc-5 netrin receptor B (UNC5B) in HepG2 and BEL-7404 cancer cells. Overall, our results indicated that MCA's efficacy in ABCB1- and ABCG2-overexpressing and cisplatin resistant cancer cells is due to the induction of apoptosis and cell cycle arrest in the G0/G1 phase.

## Introduction

Multidrug resistance (MDR) is one of the major factors that mediates the loss of efficacy of chemotherapy in cancer patients, increasing the frequency of treatment failure (1, 2). MDR can be defined resistance to a drug that produces resistance to other structurally and functionally different drugs that are distinct from the original drug (3). Mechanisms of MDR in cancer cells include: (1) an overexpression of specific ATP binding cassette (ABC) transporters; (2) increased metabolism or biotransformation of anticancer drugs; (3) increased DNA damage response and repair; (4) evasion and resistance to apoptosis; (5) mutations in the cellular targets for anticancer drugs; (6) sequestration of drugs by cellular organelles; (7) increased tolerability to the tumor microenvironment (4–11). The overexpression of efflux pump proteins known as ABC transporters is one of the most frequent mechanisms that mediates MDR, which are present on the cell membrane of certain MDR cancer cells (4, 5).

The family of ABC transporters is known as one of the largest protein families, which includes seven subfamilies from ABCA to ABCG (5, 12, 13). They are widely expressed in the intestines, blood-brain barrier (BBB), liver, placenta and kidneys (14, 15), and mediate the transport or efflux of their physiological substrates such as lipids, sterols, porphyrins, and xenobiotics (16). Among the ABC transporter sub-families, ABCB1 (multidrug resistance 1, MDR1; P-glycoprotein, P-gp), ABCG2 (breast cancer resistance protein, BCRP; mitoxantrone resistance, MXR), ABCC10 (multidrug resistance protein 7, MRP7), and ABCC1 (multidrug resistance protein 1, MRP1) play an important role in producing MDR in cancer cells (17–19). The overexpression of ABC transporters can significantly decrease the intracellular concentration of certain anticancer drugs, thereby decreasing their therapeutic efficacy (20). The expression level of ABC transporters has been shown to be significantly correlated with the level of malignancy progression and chemotherapy efficacy (21–25). Consequently, it is important to know if a chemotherapeutic drug is a substrate for a specific ABC transporter, as its overexpression will significantly attenuate or abolish the efficacy of the drug.

Cantharidin (CTD) is a biologically active compound present in *Mylabris phalerata*, the Chinese blister beetle (26). CTD has been used in traditional Chinese medicine for more than 2000 years to treat many diseases, including cancer (27–32). Recent reports indicate that CTD has significant anticancer efficacy in different types cancer cells (33–39). However, the use of CTD in clinical cancer treatment is limited due its severe adverse and toxic effects (39). Therefore, several chemically modified CTD analogs have been synthesized to produce analogs that are less toxic than CTD, thereby increasing the likelihood that these compounds could be used to treat certain types of cancer (40). One of these analogs, 2,3a,7a-trimethyl-hexahydro-4,7-epoxido-isoindol-1,3-dion, or methyl-cantharidimide (MCA), has been shown to have anticancer efficacy but was less toxic than CTD (41, 42). Currently, however, MCA's anticancer efficacy in drug resistant cancer cells, as well as its mechanism of action, remain to be elucidated.

Therefore, in this study, we determined the efficacy of MCA in ABCB1- and ABCG2-overexpressing cancer cells and in HEK293 cells transfected with either ABCB1 or ABCG2 cDNA. In addition, we also determined the efficacy of MCA in the cisplatin-resistant cancer cell lines, KCP-4 and BEL-7404/CP20. Finally, we ascertained the effect of MCA on (1) apoptosis, (2) the cell cycle, and (3) gene expression.

## Results

### The Efficacy of Paclitaxel and MCA in KB-C2 Cells That Overexpressing ABCB1 Transporter and Parental KB-3-1 Cells

**Figure 1:**
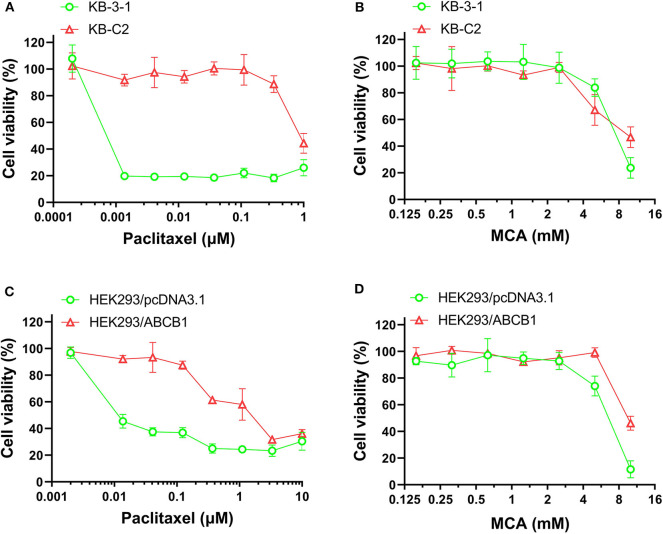
The viability of parental cells and ABCB1-overexpressing cell lines affected by MCA and paclitaxel. Concentration-dependent viability curves for the efficacy of **(A)** paclitaxel in KB-3-1 and KB-C2 cells; **(B)** MCA in KB-3-1 and KB-C2 cells; **(C)** paclitaxel in HEK293/pcDNA3.1 and HEK293/ABCB1 cell; **(D)** MCA in HEK293/pcDNA3.1 and HEK293/ABCB1 cells.

As shown in Figures 1A,B and Table 1, the efficacy of paclitaxel, a substrate of the ABCB1 transporter (43), was signfiicantly decreased (920-fold) in KB-C2 cells overexpressing the ABCB1 transporter, compared to the wild type parental cells, KB-3-1. However, the efficacy of MCA was not significantly different between KB-3-1 and KB-C2 cancer cells, indicating that MCA was efficacious in the ABCB1-overexpressing cell line, i.e., there was no resistance to MCA.

**Table 1:** The efficacy of paclitaxel and MCA in KB-3-1 cells overexpressing the ABCB1 transporter and KB-C2 cancer cells.

**Cell line**	**IC**_****50****_ **±** **SD**^****a****^ **[RF**^****b****^**]**	
	**Paclitaxel (μM)**	**MCA (mM)**
KB-3-1	0.001 ± 0.001 [1.0]	7.234 ± 0.747 [1.0]
KB-C2	0.920 ± 0.040 [920]^*^	8.435 ± 1.276 [1.17]

a *IC_50_ values were investigated and calculated using the MTT assay and were calculated based on three independent experiments*.

b *Resistant fold (RF) was calculated by dividing the IC_50_ values for MCA or paclitaxel in the KB-C2 drug resistant cells by the IC_50_ values for MCA or paclitaxel in the KB-3-1 parental (non-resistant) cells*.

* *p < 0.05*.

### The Efficacy of Paclitaxel in HEK293 Cells Transfected With the ABCB1 Gene and in HEK293 Cells Transfected With an Empty Vector

Since the resistance of KB-C2 could be affected by mutiple factors, we determined the efficacy of paclitaxel and MCA in the with ABCB1-gene-transfected cell line, HEK293/ABCB1, whose resistance was due to the overexpression of the ABCB1 transporter. As shown in Figures 1C,D, and Table 2, the efficacy of paclitaxel in HEK293 cells tranfected with the ABCB1 gene was sigficantly decreased (135.6-fold) compared to HEK293 cells that did not have the ABCB1 gene (i.e., empty vector cells). These results indicate that the HEK293 cells transfected with the ABCB1 gene overexpressed the ABCB1 transporter, although to a lesser extent, than the colchicine-selected KB-C2 cell line. Similar to the results obtained with the KB-C2 and KB-3-1 cell lines, the efficacy of MCA was not signfiicantly alterd in the HEK293 cells overexpressing the ABCB1 transporter compared to the non-overexpressing empty DNA vector transfected HEK293 cells.

**Table 2:** The efficacy of paclitaxel and MCA in HEK293 cells overexpressing the ABCB1 transporter and in HEK293 cells transfected with an empty DNA vector.

**Cell line**	**IC**_****50****_ **±** **SD**^****a****^ **[RF**^****b****^**]**	
	**Paclitaxel (μM)**	**MCA (mM)**
HEK293/pcDNA3.1	0.009 ± 0.004 [1.0]	6.370 ± 0.416 [1.0]
HEK293/ABCB1	1.220 ± 0.480 [135.56]^*^	7.999 ± 1.289 [1.26]

a *IC_50_ values were investigated and calculated using the MTT assay and were calculated based on three independent experiments*.

b *Resistance fold (RF) was calculated by dividing the IC_50_ values for MCA or paclitaxel in the HEK293 cells overexpressing the ABCB1 transporter (drug resistant) by the IC_50_ values for MCA or paclitaxel in the HEK293 cells transfected with an empty DNA vector (non-resistant) cells*.

* *p < 0.05*.

### The Efficacy of Mitoxantrone and MCA in NCI-H460/MX20 Cells Overexpressing the ABCG2 Transporter and Parental NCI-H460 Cancer Cells

**Figure 2:**
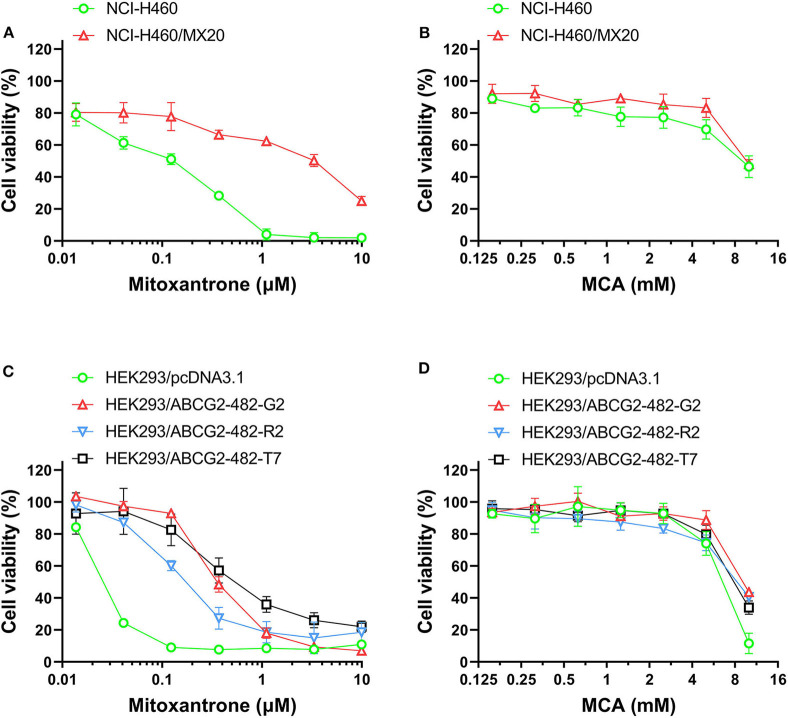
The viability of parental cells and ABCG2-overexpressing cell lines affected by MCA and mitoxantrone. The concentration-dependent viability data for the efficacy of: **(A)** mitoxantrone efficacy in NCI-H460 and NCI-H460/MX20 cells; **(B)** MCA in NCI-H460 and NCI-H460/MX20 cells; **(C)** mitoxantrone in HEK293/pcDNA3.1, HEK293/ABCG2-482-R2, HEK293/ABCG2-482-G2, and HEK293/ABCG2-482-T7 cells; and **(D)** MCA efficacy in HEK293/pcDNA3.1, HEK293/ABCG2-482-G2, HEK293/ABCG2-482-R2, and HEK293/ABCG2-482-T7.

As shown in Figures 2A,B and Table 3, the efficacy of mitoxantrone, a substrate of ABCG2 transporter (44), was significantly decreased (22-fold) in mitoxantrone-selected NCI-H460/MX20 cancer cells overexpressing the ABCG2 transporter, compared to its parental cell line, NCI-H460. In contrast, the efficacy of MCA was not significantly different (1.14-fold) in the NCI-H460 and NCI-H460/MX20 cancer cells, demonstrated that ABCG2-overexpressing cell line did not confer resistance to MCA.

**Table 3:** The efficacy of mitoxantrone and MCA in NCI-H460/MX20 cells overexpressing the ABCG2 transporter and NCI-460 parental cells.

**Cell line**	**IC**_****50****_ **±** **SD**^****a****^ **[RF**^****b****^**]**	
	**Mitoxantrone (μM)**	**MCA (mM)**
NCI-H460	0.170 ± 0.080 [1.0]	8.532 ± 0.523 [1.0]
NCI-H460/MX20	3.750 ± 0.480 [22.06]^*^	9.699 ± 0149 [1.14]

a *IC_50_ values were investigated and calculated using the MTT assay and were calculated based on three independent experiments*.

b *Resistance fold (RF) was calculated by dividing the IC_50_ values for mitoxantrone or MCA in the NCI-H460/MX20 drug resistant cells by the IC_50_ values for mitoxantrone or MCA in the KB-3-1 NCI-H460 (non-resistant) cells*.

* *p < 0.05*.

### The Efficacy of Mitoxantronre in HEK293 ABCG2 Gene Transfected Cells and in Empty DNA Vector Transfected HEK293 Cells

As shown in Figures 2C,D and Table 4, the efficacy of mitoxantrone was significantly decreased in the ABCG2-gene-transfected cell lines HEK293/ABCG2-482-R2, HEK293/ABCG2-482-G2, HEK293/ABCG2-482-T7 by 9.32-, 14.92-, and 19.49-fold, respectively, compared to the empty vector transfected cell line, HEK293/pcDNA3.1. However, the efficacy of MCA in the ABCG2-gene-transfected cells was not significantly different than the non-overexpressing empty vector transfected cells.

**Table 4:** The efficacy of mitoxantrone and MCA in the ABCG2-gene-transfected HEK293 cell lines and the HEK293 cells transfected with an empty DNA vector.

**Cell line**	**IC**_****50****_ **±** **SD**^****a****^ **[RF**^****b****^**]**	
	**Mitoxantrone (μM)**	**MCA (mM)**
HEK293/pcDNA3.1	0.059 ± 0.038 [1.0]	6.370 ± 0.416 [1.0]
HEK293/ABCG2-482-R2	0.550 ± 0.360 [9.32]^*^	8.694 ± 0.688 [1.36]
HEK293/ABCG2-482-G2	0.880 ± 0.510 [14.92]*	7.990 ± 0.931 [1.25]
HEK293/ABCG2-482-T7	1.150 ± 0.480 [19.49]*	6.828 ± 1.448 [1.07]

a *IC_50_ values were investigated and calculated using the MTT assay and were calculated based on three independent experiments*.

b *Resistance fold (RF) was calculated by dividing the IC_50_ values for mitoxantrone or MCA in the ABCG2-gene transfected HEK293 cell lines (drug resistant) by the IC_50_ values for paclitaxel or MCA in the HEK293 cells transfected with an empty DNA vector (non-resistant) cells*.

* *p < 0.05*.

### The Efficacy of Cisplatin and MCA in Cisplatin—Resistant Cancer Cells

**Figure 3:**
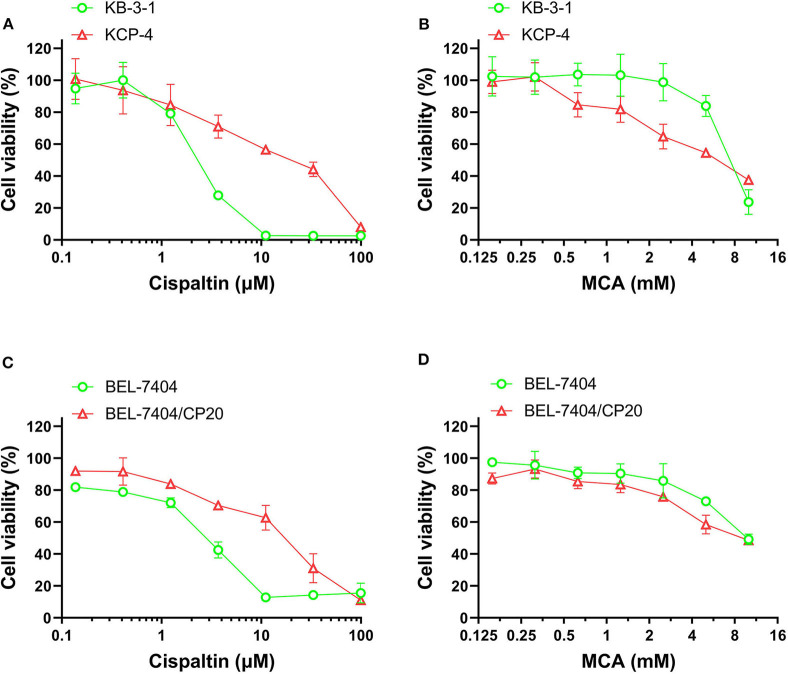
The effect of MCA and cisplatin on the viability of parental cells and cisplatin—resistant cell lines. Concentration-dependent viability curves for the efficacy of; **(A)** cisplatin in KB-3-1 and KCP-4 cells; **(B)** MCA in KB-3-1 and KCP-4 cells; **(C)** cisplatin in BEL-7404 and BEL-7404/CP20 cells; and **(D)** MCA in BEL-7404 and BEL-7404/CP20 cells.

We also determined the anticancer efficacy of MCA in the cisplatin-resistant cancer cell lines, BEL-7404/CP20 and KCP-4. The efficacy of cisplatin was significantly decreased in BEL-7404/CP20 and KCP-4 cells, compared to the parental cell lines, KB-3-1 (9.77-fold) and BEL-7404 (6.27-fold, respectively) (Figure 3 and Table 5). However, there was no significant difference in the anticancer efficacy of MCA in any of the cisplatin-resistant or parental cancer cell lines (Figure 3 and Table 5).

**Table 5:** The efficacy of cisplatin and MCA in the cisplatin-resistant cell lines, KCP-4 and BEL-7404/CP20, and their respective parental cells lines, KB-3-1 and BEL-7404.

**Cell line**	**IC**_****50****_ **±** **SD**^****a****^ **[RF**^****b****^**]**	
	**Cisplatin (μM)**	**MCA (mM)**
KB-3-1	2.930 ± 0.260 [1.0]	7.234 ± 0.747 [1.0]
KCP-4	28.620 ± 0.920 [9.77]^*^	7.052 ± 1.160 [0.97]
BEL-7404	4.800 ± 3.210 [1.0]	7.254 ± 0.478 [1.0]
BEL-7404/CP20	30.110 ± 19.860 [6.27]*	8.163 ± 1.288 [1.13]

a *IC_50_ values were investigated and calculated using the MTT assay and were calculated based on three independent experiments*.

b *Resistance fold (RF) was calculated by dividing the IC_50_ values for MCA and cisplatin for the cisplatin resistant cells by the IC_50_ values for the non-resistant parental cells*.

* *p < 0.05*.

### MCA Induced Apoptosis in BEL-7404 and BEL-7404/CP20 Cells

**Figure 4:**
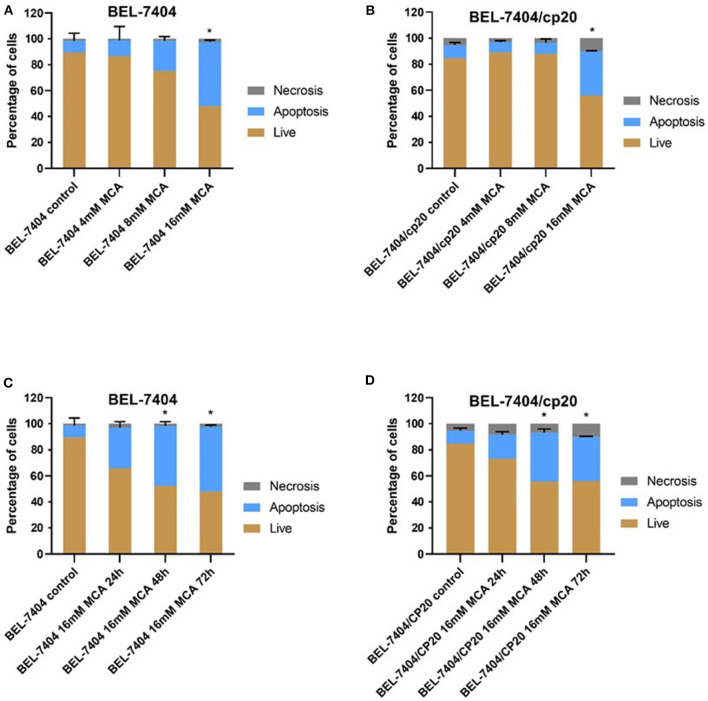
The effect of MCA on the apoptosis of BEL-7404 and BEL-7404/CP20 cells. BEL-7404 **(A)** and BEL-7404/CP20 **(B)** cells were incubated with MCA (4, 8, or 16 mM) for 72 h before test. We also incubated BEL-7404 **(C)** and BEL-7404/CP20 cells **(D)** with 16 mM of MCA for 24, 48, and 72 h. *p < 0.05 compared with the control group.

To determine if the anticancer efficacy of MCA was due to the induction of apoptosis, BEL-7404 and BEL-7404/CP20 cells were incubated with MCA (4, 8, and 16 mM) for 72 h. As shown in Figures 4A,B, the total apoptotic rate was significantly increased in in both cell lines after incubation with 16 mM of MCA. We also determined if the apoptotic-inducing effect of MCA was time-dependent by incubating BEL-7404 and BEL-7404/CP20 cells with 16 mM of MCA for 24, 48, or 72 h (Figures 4C,D). The apoptotic rate was significantly increased in BEL-7404 and BEL-7404/CP20 cells incubated with 16 mM of MCA for 48 and 72 h compared to cells incubated with vehicle.

### MCA Arrested BEL-7404 and BEL-7404/CP20 Cancer Cells in the G0/G1 Phase

**Figure 5:**
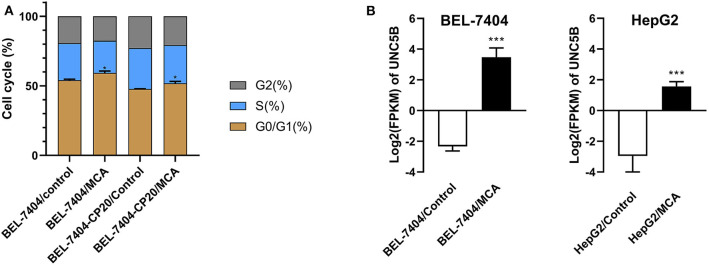
**(A)** The effect of MCA on the cell cycle of BEL-7404 and BEL-7404/CP20 cells. BEL-7404 and BEL-7404/CP20 cells were incubated with 5 mM of MCA for 48 h before test. *p < 0.05 compared with the control group. **(B)** The effect of MCA on UNC5B expression in BEL-7404 and HepG2 cells. The values of log2 fragments per kilobase million (FPKM) indicated the expression levels of UNC5B in BEL-7404 and HepG2 cells (***P < 0.001).

To elucidate the mechanism of MCA's anticancer efficacy, we determined the effect of MCA on the progression of the cell cycle in BEL-7404 and BEL-7404/CP20 cells. The progression of the cell cycle from the G0/G1 to S phase was blocked following the incubation of BEL-7404 and BEL-7404/CP20 cells with MCA (5 mM) (Figure 5 and Table 6). The percentage of cells in the G0/G1 phase incubated with MCA were significantly greater than BEL-7404 and BEL-7404/CP20 cells incubated with vehicle (*p* < 0.05). However, there was no significant difference in the percentage of cells in the G2 and S phases between MCA and vehicle in both cell lines.

**Table 6:** The effect of 5 mM of MCA on the cell cycle of BEL-7404 and BEL-7404/CP20 cell lines.

**Cell line**	**Treatment**	**G0/G1 (%)**	**S (%)**	**G2 (%)**
BEL-7404	Control	54.07 ± 0.73	26.52 ± 0.71	19.41 ± 0.32
	MCA	59.22 ± 1.43^*^	23.14 ± 1.56	17.64 ± 0.13
BEL-7404-CP20	Control	47.67 ± 0.26	29.42 ± 0.71	22.92 ± 0.45
	MCA	51.80 ± 1.40^*^	27.48 ± 1.17	20.72 ± 0.23

*Data were collected based on three independent experiments. Data are presented as mean ± Standard Deviation*.

* *p < 0.05*.

### MCA Induced the Expression of UNC5B in BEL-7404 and HepG2 Cells

To identify the underlying target and mechanism of MCA-induced apoptosis of the cancer cells used in our study, we incubated two hepatic cancer cell lines, BEL-7404 and HepG2, with 8 mM of MCA for 72 h, and then performed a differentially expressed genes (DEGs) analysis. The results indicated that the protein, UNC5B (p53RDL1), a p53 receptor required for apoptosis (45), was significantly increased in cells incubated with 8 mM of MCA compared to the control group (Figure 5B and Table 7), as indicated by the significant increase in the values of log2 fragments per kilobase million (FPKM) of UNC5B in BEL-7404 and HepG2.

**Table 7:** The effect of 8 mM of MCA on the expression level of UNC5B in BEL-7404 and HepG2 cell lines.

**Cell line/treatment**	**BEL-7404/Control**	**BEL-7404/MCA**
Log_2_(FPKM^a^)	−2.33 ± 0.30	3.48 ± 0.60***
Log_2_Fold	0	5.34***
**Cell line/treatment**	**HepG2/Control**	**HepG2/MCA**
Log_2_(FPKM^a^)	−2.95 ± 2.52	1.57 ± 0.31***
Log_2_Fold	0	2.91***

a Values of log_2_ fragments per kilobase million (FPKM) indicates the expression levels of UNC5B in BEL-7404 and HepG2 cells. Values are means ± SDs of two samples. (***P < 0.001).

## Discussion

As previously reported, the anticancer drug paclitaxel, a substrate for the ABCB1 transporter (43), inhibited the *in vitro* growth of the non-drug resistant, parental KB-3-1 cancer cells, whereas paclitaxel was much less potent (920-fold less) in the ABCB1 overexpressing KB-C2 cancer cells that are resistant to paclitaxel. In contrast, the IC_50_ values of MCA were not significantly different for KB-3-1 (IC_50_ value of 7.23 mM) and KB-C2 (IC_50_ value of 8.43 mM) cells. Similarly, paclitaxel was significantly less efficacious in ABCB1 gene transfected HEK293 cells (135-fold less), which overexpress the ABCB1 transporter (46), compared to HEK293 cells transfected with an empty DNA vector that do not overexpress ABCB1 transporter. Furthermore, there was no significant difference in the potency of MCA in the HEK293 cell lines. Our results suggest that the overexpression of the ABCB1 transporter does not confer resistance to MCA and that it has efficacy in MDR KB-C2 cells. Currently, it is unknown as to whether the above-mentioned concentrations of MCA can be obtained in human plasma without producing severe adverse or toxic effects. However, it has been shown that *in vitro*, MCA is significantly less toxic in normal human liver cells compared to hepatocellular carcinoma cells (42). Additional *in vitro*, as well as *ex-vivo* studies, will be required to fully determine the toxicological profile of MCA.

The results of our *in vitro* study also indicated, as previously reported (47), mitoxantrone, an ABCG2 substrate (44), inhibited the growth of the non-MDR cell line, NCI-H460. However, mitoxantrone was significantly less efficacious in inhibiting the growth of NCI-H460/MX20 cells, which have been shown to be resistant to mitoxantrone caused by ABCG2 transporter overexpression (47), compared with its parental cell line, NCI-H460. Similar to the results we obtained in the ABCB1 overexpressing cell lines, there was no significant difference in the IC_50_ values for MCA in NCI-H460 (8.53 mM) and NCI-H460/MX30 cells (9.69 mM). Furthermore, the efficacy of mitoxantrone was significantly decreased in HEK293/ABCG2-482-R2, HEK293/ABCG2-482-G2, and HEK293/ABCG2-482-T7 by 9.32-, 14.92-, and 19.49-fold, respectively, compared to HEK-293 cells that express either wild-type and mutant ABCG2. The ABCG2-482-R2 protein is the wild-type protein and it is overexpressed in the transfected cells, whereas the ABCG2 G2 and T7 proteins in the transfected cells overexpress the ABCG2 transporter and have alterations in their substrate and antagonist specificity due to mutations at amino acid 482 (48). Therefore, as previously shown, mitoxantrone was significantly less efficacious in cells transfected with these ABCG2 mutations. In contrast, there was no significant difference in the efficacy of MCA in the ABCG2-R2 (wild-type), ABCG2-G2 and -T7 transfected cells (IC_50_ values of 8.69, 7.99, and 6.83 mM, respectively). The results suggest that the overexpression of the ABCG2 transporter in cancer cells used in this study does not confer resistance to MCA.

We also determined the effect of MCA in cancer cells resistant to cisplatin. Similar to previous studies (49–51), the efficacy of cisplatin was significantly lower in the cisplatin-resistant cell lines, BEL-7404/CP20 and KCP-4, compared to their respective, non-cisplatin resistant parental cell lines, BEL-7404 and KB-3-1 cells. Previous *in vitro* studies have reported that cisplatin resistance in BEL-7404 cells can result from: (1) a significant increase in tolerance to DNA damage (52, 53) and (2) a decrease in the intracellular levels of cisplatin due to the overexpression of efflux proteins and a decrease in the endocytosis of cisplatin (54, 55). Cancer cells can also become resistant to chemotherapeutic drugs by decreasing or abrogating their death by apoptosis (6). Indeed, our *in vitro* results indicated that the incubation of BEL-7404/CP20 cells, as well as BEL-7404 cells, with 16 mM of MCA for 48 h, significantly increased the percentage of apoptotic cells. Additionally, the incubation of BEL-7404 and BEL-7404/CP20 cells with 5 mM of MCA significantly increased the percentage of cells that were arrested in the G0/G1 phase compared to cells incubated with vehicle. The fact that more cells were arrested in the G0/G1 phase suggests that there will be a lower number of cells that can enter the S phase of the cell cycle, thereby decreasing cellular replication. Thus, MCA inhibits the replication of BEL-7404 and BEL-7404/CP20 cancer cells.

We subsequently conducted experiments to determine the mechanism by which MCA induces the apoptosis in BEL-7404 and HepG2 cells using a differential expression gene (DEG) analysis. This approach can reveal what genes are significantly upregulated or downregulated following exposure to MCA. Our results indicated that the incubation of BEL-7404 and HepG2 cancer cells with 8 mM of MCA significantly increased the expression of the protein, UN5CB, by 5.34- and 2.91- Log_2_Fold, respectively. UNC5B, also known as the p53-regulated receptor for death and life protein (p53RDL1), is a protein that interacts with the p53 regulated receptor to induce apoptosis (45, 56). It is well-established that the p53 protein plays a key role in tumor suppression and at least 50% of all human cancers have mutations in the p53 gene (57). Furthermore, p53 mediates cell cycle arrest and cell death depending on the context of the cellular environment (58). One of the proteins involved in p53—dependent apoptosis is the netrin receptor family (56). Furthermore, UNC5B is one of netrin-1 receptor family (UNC5A to UNC5D) (59) members that participates in p53-dependent apoptosis as a target gene for p53 via a p53-binding sequence located within its intron 1 gene (45). There is a death domain in UNC5B's intracellular region that serves as a dependence receptor, i.e., the apoptotic effect of UNC5B will be inhibited when it interacts with its biological ligand, netrin-1 (56). A caspase-cleavage sequence, DXXD, which is cleaved by caspase-3 and, further induces cellular apoptosis, is present in UNC5B's intracellular region (45). The genotype of p53 in both BEL-7404 and HepG2 cells are wild-type (60, 61), indicating that the expression of UNC5B in these two cell lines was not affected by p53 mutations. Interestingly, a recent study reported that the incubation of HepG2 cells with MCA significantly upregulated caspase-3 expression, which plays a key role in apoptosis (42). Thus, in our study, the significant *in vitro* increase in UNC5B expression levels produced by MCA suggests that the increase in the apoptosis of BEL-7404 and HepG2 cells may be due to its activation of the p53-UNC5B apoptosis pathway. In addition, the arrest of the cell cycle of BEL-7404 and BEL-7404/CP20 cells produced by MCA may also be due to its effect on the p53-UNC5B axis. However, additional detailed studies must be conducted to determine how MCA induces the upregulation of UNC5B in cisplatin-resistant BEL-7404 cancer cells.

## Materials and Methods

### Chemicals

Methyl-Cantharidimide (MCA) was obtained as a gift from Sihuan Bioengineering Co., Ltd. (Jiangsu, China). Dulbecco's modified Eagle's Medium (DMEM), Bovine serum albumin (BSA), penicillin/streptomycin and 0.25% trypsin, fetal bovine serum (FBS) were purchased from Corning Incorporated (Corning, NY). Dimethylsulfoxide (DMSO), 3-(4,5-dimethylthiazol-yl)-2,5-diphenyltetrazolium bromide (MTT), paclitaxel, cisplatin, mitoxantrone were purchased from Sigma-Aldrich (St. Louis, MO). Propidium Iodide (PI) and annexin V were purchased from BD biosciences (San Jose, CA). All the rest chemicals were purchased from Sigma Chemical Co (St. Louis, MO).

### Cell Lines and Cell Culture

The ABCB1-overexpresing experiments were conducted using the human epidermoid carcinoma cell line, KB-3-1, and its ABCB1-overexpressing, colchicine-selected cells, KB-C2. For the experiments with the ABCG2-overexpresing cells, we used the non-small cell lung cancer (NSCLC) cell line, NCI-H460, and its ABG21-overexpressing, mitoxantrone-selected cells, NCI-H460/MX20. The KB-C2, and NCI-H460/MX20 cells were cultured and maintained as previously reported (47). HEK293/pcDNA3.1, HEK293/ABCB1, HEK293/ABCG2-482-R2, HEK293/ABCG2-482-G2, and HEK293/ABCG2-482-T7 cells were generated by the transfection of an empty vector pcDNA3.1, a pcDNA3.1 vector containing a full length ABCB1 gene or a pcDNA3.1 vector containing a full length ABCG2 gene, with different amino acids at position 482, including threonine, arginine, or glycine (46, 48). G418 (2 mg/ml), with complete culture medium, was used to select the transfected cells. The cisplatin resistant cell line, of KCP-4, a human hepatocellular carcinoma cell line, BEL-7404, and the cisplatin resistant cell line, BEL-7404/CP20, were used in the cisplatin resistance experiments (49–51). KCP-4 cells were maintained in 7 μg/ml cisplatin and BEL-7404/CP20 were maintained in 20 ng/ml of cisplatin with complete DMEM medium. All of the cells were cultured and maintained in DMEM medium containing 1% penicillin/streptomycin, 10% FBS and in a water jacketed CO_2_ incubator (5% CO_2_) with thermal protection. All cells were cultured under non-treatment condition for at least 14 days before testing.

### MTT Assay

Cell viability and resistance - fold were calculated using the MTT assay as previously reported (62). For the resistance experiments, the cells were digested by 0.25% trypsin-EDTA, resuspended and seeded into a 96-well plate as 5000 cells each well in 180 μL of complete medium. After incubating overnight, MCA and other anticancer drugs were added at different concentrations to produce a final volume of 200 μL. After 72 h incubation, the MTT solution (4 mg/ml) was added to each well and the plates were incubated for another 4 h. After the incubation, the supernatant was removed and 100 μL of DMSO was added and the formazan crystals were dissolved by shaking the plates for 10 min. Finally, the absorbance at 570 nm was measured using a microplate reader (Dynex Technologies, Chantilly, VA). The concentration required to inhibit cell viability by 50% (IC_50_) was calculated after plotting survival curves using the Bliss method as previously described (63).

### Apoptosis Analysis

BEL-7404 and BEL-7404/CP20 cells were incubated with MCA (4, 8, and 16 mM) for 72 h. Cell were harvested and dyed using Propidium Iodide (PI) and annexin V. The apoptosis analysis was conducted using FL-1 and FL-2 of the BD Accuri™ C6 flow cytometer as described previously (64).

### Cell Cycle

BEL-7404 and BEL-7404/CP20 cells were incubated with 5 mM of MCA for 48 h. Cell were harvested and fixed using 70% ice cold ethanol, then dyed using Propidium Iodide (PI). The cell cycle analysis was determined using a BD Accuri™ C6 flow cytometer as described previously (65).

### Differentially Expressed Genes (DEGs) Analysis

BEL-7404 and HepG2 cells were incubated with 8 mM of MCA 72 h. Cells were digested, harvested, and RNA extraction was performed using a RNA extraction kit provided by QIAGEN. All the expressed genes (DEGs) analysis data were generated and analyzed by Novogene Corporation Inc. (Sacramento, CA).

### Statistical Analysis

All data were collected based on at least three independent experiments. The data were analyzed using a one-way analysis of variance (ANOVA) and *post-hoc* tests were done a *t*-test method. The *a priori* significance level was *p* < 0.05.

## Data Availability Statement

The raw data supporting the conclusions of this article will be made available by the authors, without undue reservation.

## Author Contributions

Y-DL: data curation. Y-DL, X-DD, Z-NL, and YY: formal analysis, investigation, and visualization. Y-DL, YM, CA, D-HY, Y-FF, and Z-SC: methodology. Y-FF and Z-SC: project administration. YM, LL, D-HY, and Z-SC: resources. Z-SC: supervision. Y-DL, YM, X-DD, Z-NL, YY, and LL: validation. Y-DL and YM: writing—original draft. Y-DL, YM, X-DD, Z-NL, YY, CA, D-HY, Y-FF, and Z-SC: writing—review and editing. All authors have read and agreed to the published version of the manuscript.

## Conflict of Interest

The authors declare that the research was conducted in the absence of any commercial or financial relationships that could be construed as a potential conflict of interest.
